# Extended-release naltrexone/bupropion is safe and effective among subjects with type 2 diabetes already taking incretin agents: a post-hoc analysis of the LIGHT trial

**DOI:** 10.1038/s41366-021-00831-4

**Published:** 2021-06-03

**Authors:** Sean Wharton, Peter Yin, Melonie Burrows, Errol Gould, Jessica Blavignac, Rebecca A. G. Christensen, Elham Kamran, Fernando Camacho, Maxime Barakat

**Affiliations:** 1 The Wharton Medical Clinic, Toronto, ON Canada; 2 Bausch Health, Laval, QC Canada; 3 Currax Pharmaceuticals LLC, Morristown, NJ USA; 4 Department of Statistics and Actuarial Sciences, University of Waterloo, Waterloo, ON Canada

**Keywords:** Weight management, Randomized controlled trials, Diabetes

## Abstract

**Background:**

Extended-release naltrexone/bupropion (NB) is indicated for chronic weight management. Incretin agents are recommended for patients with type 2 diabetes. This analysis looked at the add-on of NB to incretins to see if weight loss could occur in patients already stabilized on incretin agents.

**Methods:**

This was a post-hoc analysis of NB vs. placebo (PL) among subjects with type 2 diabetes stable on an incretin agent prior to randomization in a double-blind, PL-controlled cardiovascular outcome trial (*N* = 1317).

**Results:**

Over 1 year, mean weight loss was significantly greater among NB patients vs. PL among those taking DPP-4i (mean absolute difference 4.6% [*p* < 0.0001]) and those taking GLP-1RAs (mean absolute difference 5.2%, *p* < 0.0001). Proportions of subjects achieving 5% weight loss were significantly greater for NB vs. PL at weeks 26 and 52 among those taking DPP-4is or GLP-1RAs. There were no significant differences in effectiveness observed between NB + DPP-4i and NB + GLP-1RA or between PL + DPP-4i and PL + GLP-1RA in any of the analyses. Serious adverse events were reported by 9.1% and 11.1% for PL + DPP-4i and PL + GLP-1RA, respectively, and 13.3% and 12.4% of NB + DPP-4i and NB + GLP-1RA, respectively.

**Conclusion:**

NB appears to be effective in reducing weight in patients with T2DM and obesity/overweight who are taking DPP-4ihibitors or GLP-1RA. The SAE rates in all arms of this analysis were lower than have been reported in other cardiovascular outcome trials in type 2 diabetes.

## Introduction

Obesity is highly prevalent among individuals with type 2 diabetes; it is estimated that 80–90% of people with type 2 diabetes have obesity or overweight [1]. Guidelines for the management of type 2 diabetes, such as those from Diabetes Canada (2018) state that attaining and maintaining a healthy body weight, and preventing weight regain, are key components of optimizing glycemic control in people with diabetes [2].

The combination of extended-release naltrexone + bupropion (NB) is a medication approved in the US, Canada and many other countries for chronic weight management, including among patients with type 2 diabetes. The efficacy and safety of this combination have been demonstrated in four 56-week, placebo (PL)-controlled, randomized studies, including one conducted entirely in patients with type 2 diabetes (the COR-DM study) [3–6]. In the COR-DM study, 505 patients were treated with standardized lifestyle intervention and randomized 2:1 to NB or PL. NB resulted in a significantly greater weight reduction (5.0% vs. 1.8%; *p* < 0.001) and proportion of patients achieving at least a 5% weight loss (44.5% vs. 18.9%, *p* < 0.001) compared with PL [6]. NB was also associated with significantly greater reductions in A1C vs. PL (−0.6% vs. −0.1%, *p* < 0.001) and a significantly higher proportion of patients achieving A1c of <7.0% (44.1% vs. 26.3%; *p* < 0.001). In terms of safety and tolerability, NB was associated with a higher incidence of nausea (42.3% vs. 7.1%), constipation (17.7% vs. 7.1%), and vomiting (18.3% vs. 3.6%) compared to PL [6].

Many patients with type 2 diabetes are taking dipeptidyl peptidase 4 inhibitors (DPP-4is) or glucagon-like peptide-1 receptor agonists (GLP1-RAs)—both of which work through the incretin pathway in the gut [2, 7, 8]. Both of these classes of agents are associated with a low propensity to induce hypoglycemia; are either weight neutral (DPP-4is) or are associated with weight loss (GLP1-RA), and have demonstrated either neutrality or benefit in cardiovascular outcome trials (CVOTs) in type 2 diabetes [2]. As such, and also given the possibility that there could be additive weight loss with the combination of NB and GLP-1RA, it is of interest to characterize the effectiveness and safety of NB among patients with diabetes taking those medications. The LIGHT trial, the cardiovascular outcome trial (CVOT) for NB conducted from 2012 to 2015 [9], included a substantial number of patients with type 2 diabetes who were also on a GLP-1RA or a DDP-4i. This allows for a statistically robust analysis of the effectiveness and safety of NB taken for weight management among individuals with diabetes receiving DDP-4is or GLP-1RAs for glycemic management.

The objective of this post-hoc analysis of the LIGHT trial was to investigate the effectiveness and safety of NB vs. PL among patients with type 2 diabetes and overweight or obesity who report taking either a DPP-4i or a GLP1-RA.

## Methods

The subjects were drawn exclusively from the LIGHT CVOT [9]. The design of this study has been described in detail previously and is summarized in Table 1. Subjects included in the current analysis had type 2 diabetes and overweight or obesity; were on either a DPP-4i or a GLP1-RA at baseline, and had a weight change of ≤3% within three months prior to screening.

**Table 1:** Characteristics of source study: LIGHT cardiovascular outcomes trial [9].

Variable	Description
Description	Phase 3b, placebo-controlled, randomized cardiovascular outcomes trial (CVOT)
Duration	2–4 years
*N* (ITT)	8910
Population	Individuals at increased risk of adverse CV outcomes - Pre-existing CVD OR - T2DM + ≥2 of: hypertension, dyslipidemia^a^, current smoking
Age range	Men ≥45 years Women ≥50 years
BMI inclusion criteria	27–50 kg/m^2^
Other key inclusion criteria	Men: WC ≥ 102 cm Women: WC ≥ 88 cm
Randomization	1:1, NB:PL
NB dosing	Initial dose: One tablet 8/90 mg Maintenance dose: 2 tablets b.i.d. (32/360 mg)
Timing of study visits	BL, 8, 16, 26, 52, 78, 104, 130, 156, 182, and 208
Antihyperglycemic medication	Allowed, with no stated specific requirements
Other CV medications (e.g., for hypertension, dyslipidemia)	Allowed, with no stated specific requirements
Other weight loss intervention	Encouraged (but not required) to participate in an Internet-based weight management program All had access to a personal weight-loss coach, programs to track weight, meals, and physical activity; and a low-fat, low-calorie meal plan
Anthropometric data recorded	Weight (kg), height (cm), waist circumference (cm)
Primary endpoint	Time from randomization to first confirmed occurrence of a major adverse CV event (CV death, nonfatal MI, or nonfatal stroke)

*BL* baseline, *BMI* body mass index, *BP* blood pressure, *CV* cardiovascular, *CVD* cardiovascular disease, *FBG* fasting blood glucose, *HbA1c* glycated hemoglobin, *MI* myocardial infarction, *NB* extended-release naltrexone/extended-release bupropion 32/360 mg, *PL* placebo.

^a^Dyslipidemia requiring pharmacotherapy and/or high-density lipoprotein cholesterol <1.30 mmol/L (men) or <1.04 mmol/L.

In addition to the randomized treatments (NB or PL), all subjects in both arms of the LIGHT trial were enrolled in a comprehensive weight management program delivered via a web-based platform. The program was governed by a lifestyle advisory board consisting of leading experts in the field. It included internet- or telephone-based, closed-group counseling sessions led by registered dietitians, lasting 30–45 min each. They were conducted once weekly for 16 weeks, once every other week for the next eight weeks, and monthly thereafter through week 104. A meal and activity plan was also part of the weight management program. It included educational resources, weight tracking and goals, meal tracking in combination with a low-fat, calorie-counting plan, and activity tracking. Subjects were encouraged to participate in a moderately intensive exercise program.

Bodyweight (assessed to the nearest 0.1 kg) was measured at each study visit, as were height and waist circumference (in cm).

Notably, in the LIGHT trial, subjects who did not lose 2% or more of initial body weight, or who experienced a sustained increase in systolic or diastolic blood pressure (BP) of 10 mmHg or more during the first 16 weeks of randomized treatment were discontinued from study medication but were still followed in the study [9]. However, for the present analyses, the weight data for these individuals were not included after they had discontinued study medication (NB or PL).

The LIGHT trial was terminated early after the pharmaceutical company released confidential interim data. The executive steering committee recommended trial termination on May 12, 2015. The 50% interim analysis was completed on March 3, 2015 (from a database lock on February 3, 2015). Additional outcomes accumulated after the February 2015 database lock are included in a sensitivity analysis, which reports results after 64% of planned events. While the planned assessment of CV safety was compromised by early termination, there remains a large body of data on long-term weight change and maintenance that is used in the current analysis.

**Fig. 1:**
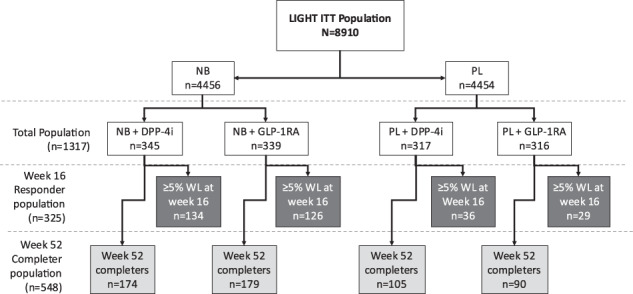
Patient disposition of populations of interest. Total population includes all patients from LIGHT who were on a DPP-4i or GLP-1RA at baseline and received study treatment. Week 52 completers are those in the total population who remained on study therapy at week 52. Week 16 responders are those in the total population who experienced a weight loss of at least 5% at week 16.

For the current publication, the four treatment groups analyzed were NB + DPP-4i, NB + GLP-1RA, PL + DPP-4i, and PL + GLP-1RA. Analyses of the data focus on three populations: total population, week 52 completers, and week 16 responders. The total population includes all patients from LIGHT who were on a DPP-4i or GLP-1RA at baseline and who received study treatment (either NB or PL). The total population includes 1317 subjects, including 684 subjects randomized to NB (51.9% of the total population) and 633 (48.1%) randomized to PL (Fig. 1). Week 52 completers are those in the total population who remained on study therapy (either NB or PL) to week 52. The completer population consisted of 548 subjects (41.6% of the total population), including 353 (64.4%) treated with NB and 195 (35.6%) treated with PL. Week 16 responders are those in the total population who experienced a weight loss of at least 5% at study week 16. This population consisted of 325 subjects (24.6% of the total population), including 260 (80.0%) treated with NB and 65 (20.0%) treated with PL (Fig. 1). Of the 325 week, 16 responders, 260 (80.0%) went on to be week 52 completers.

The analyses conducted in the total population include absolute and percent weight changes by treatment group at weeks 8, 16, 26, and 52; the proportion of patients with categorical 5% and 10% change in weight by treatment group at 26 and 52 weeks; and odds ratios for comparisons of treatment groups for percent weight loss at 52 weeks and categorical (5% or 10% weight loss) at 26 and 52 weeks. Comparisons were made as follows: PL + DPP-4i vs. NB + DPP-4i; PL + GLP-1RA vs. NB + GLP-1RA; PL + DPP-4i vs. PL + GLP-1RA; and NB + DPP-4i vs. NB + GLP-1RA.

In the week-52 completer and week-16 responder populations, the main analysis was the percent weight change at weeks 8, 16, 26, and 52. The same inter-group comparisons were made as those listed above.

Safety analyses (AEs leading to discontinuation and serious AEs) were conducted in the total population up to 52 weeks.

All patients provided written informed consent, and they were conducted according to the guidelines and principles of Good Clinical Practices standards and the Declaration of Helsinki.

### Statistical methodology

A mixed model using all available on-treatment data for weight change or percent change from baseline as response and, age, gender, and baseline weight treatment, week and treatment/week interaction as explanatory variables were used to analyze, respectively, the weight change or the percent change over time. The model used the identity link function and an unstructured covariance. Separate models were fitted for the total population, for the week 52 completers, and for the 16-week responders. A generalized mixed model using as a response the binary outcome of whether or not the subject achieved at least 5% (or 10%) weight loss at a visit and the variables indicated above as explanatory variables were used to analyze the proportion of subjects achieving at least 5% or 10% weight loss at 52 weeks. The model used the logit link function.

## Results

Baseline characteristics for the total population are shown in Table 2. Overall, 55.1% (*n* = 726) were women, 85.0% (1119) were White/Caucasian, and the mean age (SD) was 60.7 (7.0) years. Mean body weight at baseline was 107.0 kg (19.4) and mean BMI (SD) was 37.5 kg/m^2^ (5.35).

**Table 2:** Summary of subject baseline characteristics.

	All	Treatment				SMD
		PL		NB		
		DPP-4i	GLP-1RA	DPP-4i	GLP-1RA	
*N*	1317	317	316	345	339	–
Sex, *n* (%)						
Female	726 (55.1%)	159 (50.2%)	179 (56.6%)	195 (56.5%)	193 (56.9%)	0.068
Male	591 (44.9%)	158 (49.8%)	137 (43.4%)	150 (43.5%)	146 (43.1%)	
Grouped race, *n* (%)						
White/Caucasian	1119 (85.0%)	262 (82.6%)	278 (88.0%)	286 (82.9%)	293 (86.4%)	0.135
Black/African American	165 (12.5)	44 (13.9%)	31 (9.8%)	54 (15.7%)	36 (10.6%)	
Other or unknown	33 (2.5%)	11 (3.5%)	7 (2.2%)	5 (1.4%)	10 (2.9%)	
Age, years (SD)	60.7 (7.0)	61.3 (7.1)	60.4 (6.6)	60.9 (7.5)	60.1 (6.7)	0.101
BMI, kg/m^2^ (SD)	37.5 (5.4)	37.3 (5.1)	38.0 (5.6)	36.9 (5.2)	37.9 (5.5)	0.121
Weight, kg (SD)	107.0 (19.4)	106.2 (18.5)	109.0 (20.4)	105.1 (19.2)	107.7 (19.4)	0.116
Waist circumference, cm (SD)	120.2 (13.1)	119.5 (12.3)	121.4 (13.5)	119.2 (13.2)	121.0 (13.3)	0.102
Systolic blood pressure, mmHg (SD)	124.9 (12.7)	125.2 (12.7)	125.2 (13.2)	126.4 (12.3)	122.8 (12.6)	0.141
Diastolic blood pressure, mmHg (SD)	73.7 (8.9)	73.4 (8.5)	74.4 (8.5)	73.6 (9.0)	73.6 (9.3)	0.058
Heart rate, bpm (SD)	74.7 (10.7)	73.6 (11.0)	76.4 (10.2)	72.8 (10.4)	75.9 (10.8)	0.21
Hemoglobin A1c, % (SD)	7.5 (1.5)	7.7 (1.5)	7.5 (1.5)	7.5 (1.5)	7.4 (1.3)	0.09
Glucose at baseline, mmol/L (SD)	8.8 (3.8)	9.1 (3.8)	8.5 (3.7)	9.1 (3.7)	8.6 (3.8)	0.107

All values are means unless otherwise stated.

*BMI* body mass index, *DPP4i* dipeptidyl peptidase IV inhibitor, *GLP1 RA* glucagon-like peptide-1 receptor agonist, *NB* extended-release naltrexone/extended-release bupropion 32/360 mg, *PL* placebo, *SD* standard deviation, *SMD* standardized mean difference.

Of the 1317 subjects in the total population, 737 (56%) achieved at least a 2% weight loss at week 16. By LIGHT study protocol, the remaining 580 had their study medication discontinued. For the subjects who discontinued study medication at week 16, data before medication discontinuation was included in the analysis, while data after medication discontinuation was excluded.

**Fig. 2:**
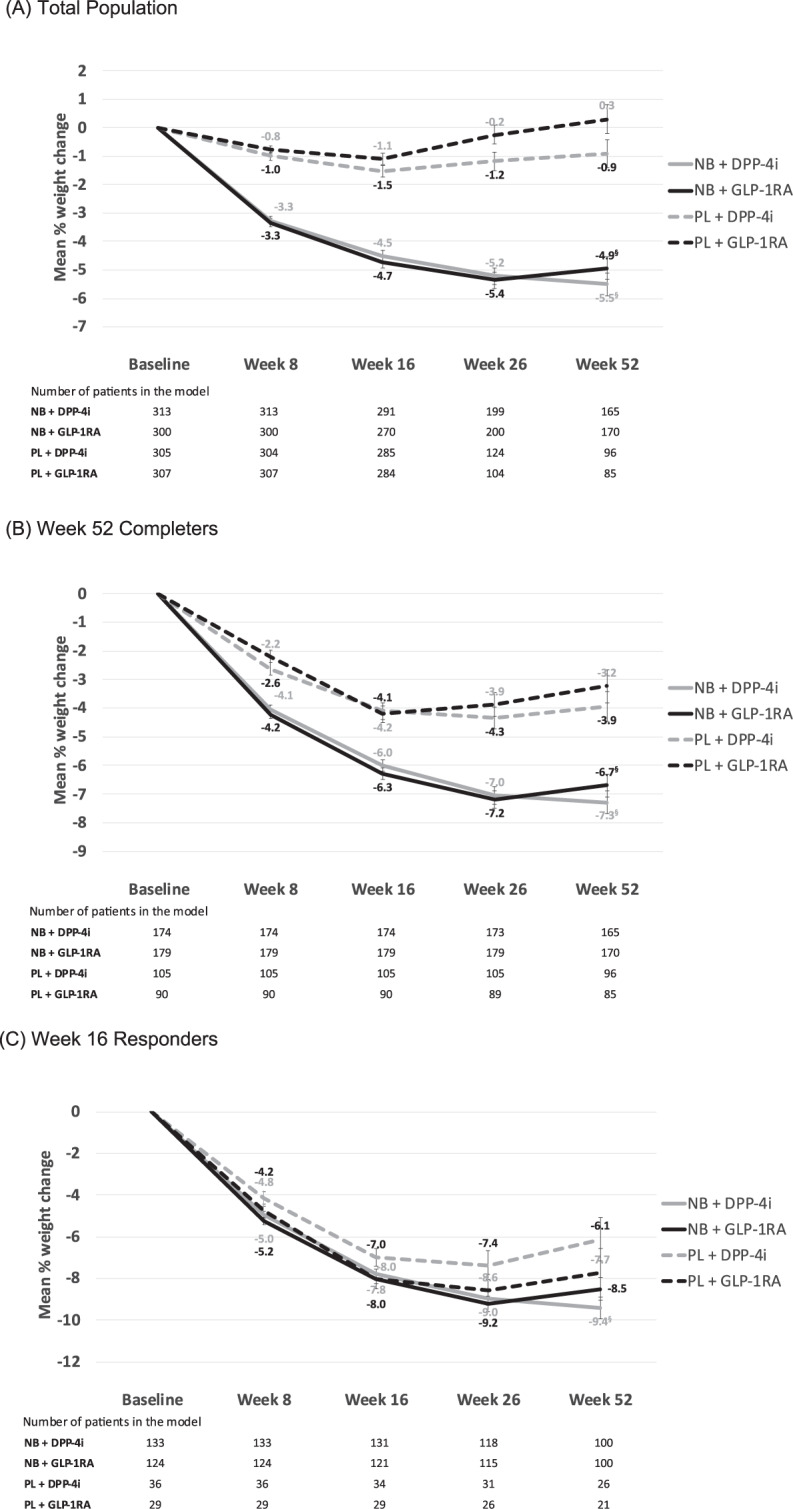
Percent weight changes. **A** Total population*, **B** week 52 completers^†^; **C** week 16 responders^‡^. ^§^Statistically significant difference, NB vs. PL at week 52. *All subjects taking a DPP-4i or GLP-1RA at baseline. ^†^Subjects from the total population who remained on study treatment through to week 52. ^‡^Subjects from the total population who had a weight loss of ≥5% from baseline at week 16. The number of subjects in the model is lower than at baseline since not all subjects have post-baseline data. Note that the attrition from week 16 to week 26 in the total population includes those patients who did not achieve 2% weight loss at week 16 (discontinued from study medication as per LIGHT study protocol and not included in this analysis). Numbers discontinuing at week 16 in the total population (**A**): NB + DPP-4i, *n* = 66; NB + GLP-1RA, *n* = 53; PL + DPP-4i, *n* = 155; PL + GLP-1RA, *n* = 173.

Mean absolute weight changes (model adjusted estimates) in the total population are shown in Supplementary Table 1. Percent weight changes (model adjusted estimates) in the total population (*n* = 1317) are shown in Fig. 2A. Both NB groups were associated with significantly larger percentage weight reductions at 52 weeks than PL. Among subjects taking DPP-4is, the mean percent weight change from baseline to week 52 was −5.5% for NB and −0.9% for PL (treatment difference −4.6%, 95% CI −5.84 to −3.37, *p* < 0.0001). Among those taking GLP-1RAs, the mean weight change from baseline to 52 weeks was −4.9% for NB and +0.3% for PL (treatment difference −5.2%, 95% CI −6.51 to −3.97, *p* < 0.0001).

**Fig. 3:**
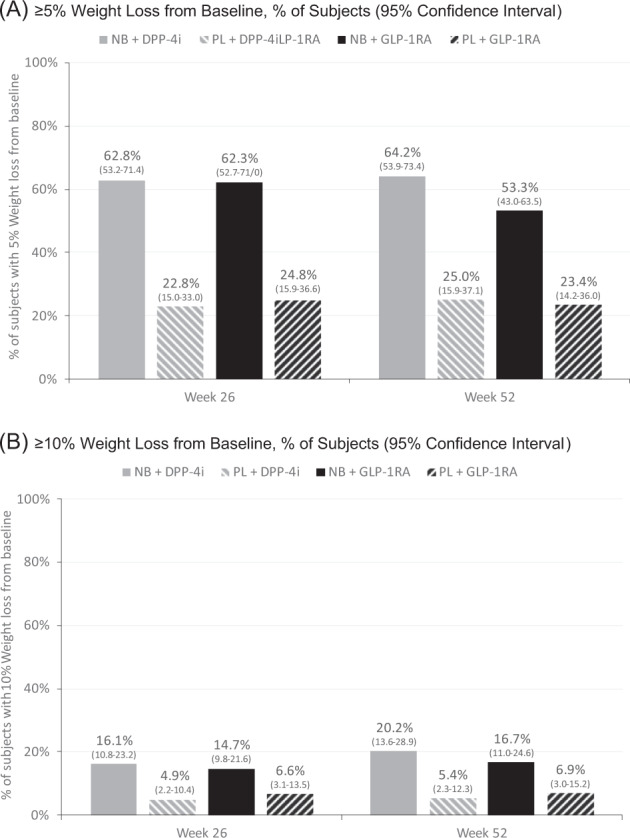
Categorical weight loss, total population at weeks 26 and 52. **A** ≥5% Weight loss from baseline; **B** ≥10% weight loss from baseline.

With respect to categorical weight loss, the proportions of subjects in each treatment group achieving 5% and 10% weight reductions from baseline to weeks 26 and 52 are shown in Fig. 3A, B. At week 26, the model adjusted proportions of achieving 5% reduction were 62.8% and 62.3% for NB + DPP-4i and NB + GLP-1RA, respectively; and 22.8% and 24.8% for PL + DPP-4i and PL + GLP-1RA, respectively. At week 52, the proportions achieving 5% weight loss were 64.2% and 53.3% for NB + DPP-4i and NB + GLP-1RA, respectively; and 25.0% and 23.4% for PL + DPP-4i and PL + GLP-1RA, respectively. The adjusted odds of achieving at least a 5% weight loss at week 26 was significantly greater for NB + DPP-4i vs. PL + DPP-4i (OR 5.71, 95% CI 3.00–10.90, *p* < 0.0001) and for NB + GLP-1RA vs. PL + GLP-1RA (OR 5.00, 95% CI 2.52–9.90, *p* < 0.0001). The odds ratios remained statistically significant at week 52 for both NB + DPP-4i vs. PL + DPP-4i (OR 5.38, 95% CI 2.63–11.00, *p* < 0.0001) and NB + GLP-1RA vs. PL + GLP-1RA (OR 3.75, 95% CI 1.78–7.87, *p* = 0.0005).

For 10% categorial weight loss at week 26, the model adjusted proportions of achieving 10% reduction were 16.1% and 14.7% for NB + DPP-4i and NB + GLP-1RA, respectively; and 4.9% and 6.6% for PL + DPP-4i and PL + GLP-1RA, respectively. At week 52, the proportions achieving 10% weight loss were 20.2% and 16.7% for NB + DPP-4i and NB + GLP-1RA, respectively; and 5.4% and 6.9% for PL + DPP-4i and PL + GLP-1RA, respectively. At week 26, the adjusted odds ratios for achieving 10% weight loss were statistically significant for the NB vs. PL comparison among those taking DPP-4is (OR 3.76, 95% CI 1.47–9.63, *p* = 0.0059), but not among those taking GLP-1RAs (OR 2.45, 95% CI 0.98–6.15, *p* = 0.0559). The week 52 analysis showed a similar pattern. At that time point, the odds of achieving a 10% weight loss were significantly greater for NB vs. PL among DPP-4i-treated subjects (OR 4.42, 95% CI 1.60–12.18, *p* = 0.0041), but the difference among GLP-1RA-treated subjects was not statistically significant (OR 2.68, 95% CI 0.99–7.27, *p* = 0.0526).

In the 52-week completer population (*n* = 548), adjusted percent weight changes at weeks 8, 16, 26, and 52 are shown in Fig. 2B. At week 52, those in the NB group had significantly larger weight changes compared to PL among those receiving DPP-4is (−7.3% for NB vs. −3.9% for PL, absolute difference −3.3%, 95% CI −4.67 to −2.03, *p* < 0.0001) and among those receiving GLP-1RAs (−6.7% for NB vs. −3.2% for PL, absolute difference −3.5%, 95% CI −4.85 to −2.10, *p* < 0.0001).

In the week 16 responder population (*n* = 322), adjusted percent weight changes at weeks 8, 16, 26, and 52 are shown in Fig. 2C. At week 52, subjects in the NB group showed significantly larger weight change compared to PL among those receiving DPP-4is, (−9.4% for NB vs. −6.1% for PL, absolute difference −3.3%, 95% CI −5.63 to −0.96, *p* = 0.0058), but the NB-PL difference among those receiving GLP-1RAs was not statistically significant (−8.5% for NB vs. −7.7% for PL, absolute difference −0.8%, 95% CI −3.31 to 1.75, *p* = 0.5459).

Across all of the effectiveness analyses described above (overall, completers, week 16 responders) there were no significant differences observed between NB + DPP-4i and NB + GLP-1RA or between PL + DPP-4i and PL + GLP-1RA.

The summary of adverse events in the total population is shown in Table 3. Serious AEs were reported by 13.3% and 12.4% of the NB + DPP-4i and NB + GLP-1RA groups, respectively; and 9.1% and 11.1% of the PL + DPP-4i and PL + GLP-1RA groups, respectively. The most frequently reported AEs in the NB + incretin groups were nausea (24/345 [7.0%] for NB + DPP-4i and 32/339 [9.4%] for NB + GLP-1RA), vomiting (5/345 [1.4%] for NB + DPP-4i and 10/339 [2.9%] for NB + GLP-1RA), constipation (7/345 [2.0%] for NB + DPP-4i and 7/339 [2.1%] for NB + GLP-1RA), unstable angina (4/345 [1.2%] for NB + DPP-4i and 8/339 [2.4%] for NB + GLP-1RA) and tremor (4/345 [1.2%] for NB + DPP-4i and 8/339 [2.4%] for NB + GLP-1RA).

**Table 3:** Adverse event summary: total population.

Adverse events	PL		NB	
	DPP4 (*n* = 317)	GLP1 (*n* = 316)	DPP4 (*n* = 345)	GLP1 (*n* = 339)
Serious AE	29 (9.1%)	35 (11.1%)	46 (13.3%)	42 (12.4%)
Severe AE	16 (5.0%)	18 (5.7%)	28 (8.1%)	32 (9.4%)
Mild AE	7 (2.2%)	12 (3.8%)	35 (10.1%)	38 (11.2%)
Study-drug-related	15 (4.7%)	9 (2.8%)	57 (16.5%)	65 (19.2%)
AEs with frequency ≥1% in any treatment group, *n* (%)				

Body system	AE	PL		NB	
		DPP4 (*n* = 317)	GLP1 (*n* = 316)	DPP4 (*n* = 345)	GLP1 (*n* = 339)
Cardiac	Unstable angina	1 (0.3%)	2 (0.6%)	4 (1.2%)	8 (2.4%)
Gastrointestinal	Constipation	–	–	7 (2.0%)	7 (2.1%)
	Diarrhea	1 (0.3%)	1 (0.3%)	2 (0.6%)	4 (1.2%)
	Nausea	2 (0.6%)		24 (7.0%)	32 (9.4%)
	Vomiting	–	–	5 (1.4%)	10 (2.9%)
General disorders	Non-cardiac chest pain	–	2 (0.6%)	1 (0.3%)	5 (1.5%)
Musculoskeletal/connective tissue	Osteoarthritis	1 (0.3%)	6 (1.9%)	–	4 (1.2%)
Nervous system	Headache	–	1 (0.3%)	–	4 (1.2%)
	Tremor	–	–	4 (1.1%)	8 (2.4%)

A separate efficacy analysis was also conducted, in which the small number of patients from the COR-DM study who were taking DPP-4is (*n* = 29) were pooled with the LIGHT study subjects. The addition of these subjects into the data set was not associated with any significant changes in the findings for any of the efficacy or safety analyses.

## Discussion

This analysis suggests that the addition of NB to a therapeutic regimen for patients with type 2 diabetes and overweight or obesity is both effective and safe for weight loss among patients who are receiving an incretin agent (either DPP-4i or GLP-1RA).

These data fill an important gap in knowledge about the concomitant use of NB with incretin agents. Although the COR-DM study would appear to be a reasonable study to examine the interaction between agents used for diabetes treatments and NB, it was conducted at a time (2007–2009) when the incretin agents were still new for the treatment of diabetes. Reflective of the antihyperglycemic medications favored during the years of the trial, metformin was used by approximately three-quarters of the subjects, nearly half were receiving a sulfonylurea (SU) and ~30% were receiving a thiazolidinedione (TZD). Only 29 COR-DM subjects were taking a DPP-4i and none were taking a GLP1-RA. Since then, SUs and TZDs have fallen out of favor, while DPP-4is, GLP1-RAs and sodium-glucose transport protein 2 inhibitors (SGLT2is) are among the add-on therapies most commonly recommended by current guidelines for type 2 diabetes [10–12].

With respect to weight loss, we noted the effectiveness of NB remains in subjects taking concomitant DPP4i or GLP1-RA therapy. In the current analysis, week 52 mean weight loss among patients in the PL group who were on a DPP4i was −0.9%, compared to −5.5% for NB (absolute difference −4.6%) and among those on a GLP1-RA, mean weight loss was +0.3% with PL and −4.9% with NB (absolute difference −5.2%). Similar results were seen in the week 52 completers and week 16 responders; where the benefit of NB relative to PL continued to be evident. The weight loss in this analysis is notable, given that the absolute difference here is >5% for some groups, a threshold of weight loss endorsed by many obesity guidelines as having a significant impact on obesity-related morbidity and mortality.

When comparing patients on GLP-1RAs with patients that were taking DPP-4is, there was no difference in weight change for those in the same randomized treatment (NB or PL). This suggests that NB maintains similar effectiveness regardless of potential previous medication-induced weight changes. Notably, however, we did not have access to data on the timing of DPP-4i/GLP1-RA initiation or weight loss history. This means that we do not know if the GLP-1RA or DPP-4i had been initiated the day before study entry, years before, or anywhere in between. Nor do we know whether or not (or to what degree) patients had experienced prior weight loss in association with the administration of their antihyperglycemic regimens. What we do know (given it was an inclusion criterion of the LIGHT study) was that subjects had not had a weight change of 3% or greater within the 3 months prior to enrolment [9]. Given that GLP-1RAs use is associated with weight loss [2], it would have been informative to know whether or not they had previously experienced GLP-1RA-related weight loss. It may still be possible that should the individuals with type 2 diabetes have initiated GLP1s at the same time as NB, they would have experienced more weight loss than those prescribed weight neutral agents such as DPP-4is, owing to a complementary or synergistic effect of the two types of weight-loss medications. It may also be that the two differing mechanisms do not have any additive effects; the design of this analysis and the nature of the source data did not allow for this to be investigated.

A safety analysis of these data was completed and notably, this is the only such analysis of NB-incretin combinations in patients with type 2 diabetes. In this analysis, there are more than 300 subjects in each of the NB + DPP4i and NB + GLP1-RA subgroups. The rates of serious AEs were 13.3% and 12.4% among those in the NB + DPP-4i and NB + GLP-1RA groups, respectively, and 9.1% and 11.1% among the PL + DPP-4i and PL + GLP-1RA groups, respectively. While the approximate 4% absolute risk difference between NB and PL among those taking DPP-4is was notable, this difference may not be clinically important given the potential for substantial benefit (PL-adjusted weight loss >3%) associated with NB-related weight loss in this analysis. It should also be considered that relative to other CVOTs among patients with type 2 diabetes, the SAE rates in all the groups of the present analysis (9.1–13.0%) are quite low. For example, in the LEADER study, which evaluated liraglutide vs. PL among patients with type 2 diabetes, SAEs occurred in 49.7% of liraglutide-treated patients and 50.4% of PL-treated patients over a median follow-up of 3.8 years [13]. In the DECLARE study, which evaluated dapagliflozin vs. PL, the SAE rates were 34.1% with dapagliflozin and 36.2% with PL over a median of 4.2 years [14]. Additionally, since our data are derived from a subpopulation of a study designed to evaluate CV outcomes and our population of interest was not prespecified by the initial LIGHT study protocol, the integrity of the randomization can no longer be assumed, and the results should therefore be interpreted with caution.

## Strengths and limitations

The strengths of this analysis are a large number of patients included (*N* = 1317) and the well-defined methodology of the source study (LIGHT [9]). However, this was a post-hoc analysis, not a prospectively defined subgroup analysis. Therefore, care should be taken in the interpretation of the results, as they should be considered exploratory rather than conclusive [15]. Examination of changes in metabolic parameters would also have been helpful in the context of this analysis. However, the LIGHT study protocol did not specify repeat measurement of blood glucose, HbA1c, or lipids during follow-up, so the data for this analysis were not available.

## Future directions

SGLT-2 inhibitors, which were approved for use in the USA starting in August 2014 [16], are widely used oral antihyperglycemic agents in type 2 diabetes. The LIGHT study ran from 2012 to 2015; as such there were very few patients taking SGLT-2 inhibitors in the study (*n* = 0 for NB and *n* = 2 for PL at baseline and *n* = 16 for NB and *n* = 18 for PL at year one; unpublished data on file, Bausch Health, Laval, QC, Canada). Research investigating the use of NB among patients with type 2 diabetes taking concurrent SGLT-2 inhibitors would be a welcome addition to the literature.

## Conclusions

This study demonstrated that NB is an effective and generally well-tolerated medication for weight loss among patients with type 2 diabetes who are on incretin agents, specifically DDP-4is and GLP-1RAs. The management of concomitant obesity and diabetes involves a multifactorial approach, including consideration of the weight effects of antihyperglycemic therapy. In this analysis, patients were already on incretin agents for glycemic management at the time of the addition of NB or PL. It may be interesting to further study the combination of NB when these incretin agents are specifically prescribed at doses for weight loss in patients with diabetes. The current analysis gives us the confidence to combine the currently available incretin diabetes medications and NB.

### Summary

#### What was known before

- Glucagon-like peptide (GLP)-1 receptor agonists (GLP1-RAs) and dipeptidyl peptidase IV inhibitors (DPP-4is) are commonly used, widely approved agents for the management of glycemia among patients with type 2 diabetes, and are associated with weight loss (GLP1-RAs) or weight neutrality (DPP-4is). Both classes of agents operate through the incretin pathway in the gut.
- Extended-release naltrexone–bupropion (NB) is a chronic weight management medication approved in many countries around the world for use in people with obesity or overweight, including individuals with type 2 diabetes.
- There are no published data available investigating the effectiveness or safety of NB use in patients with type 2 diabetes taking GLP1-RAs or DPP-4is.

#### What this study adds

- Shows weight loss effectiveness of NB among patients taking incretin agents
- Provides reassurance of the safety of NB in people with type 2 diabetes taking incretin agents (GLP1-RAs or DPP-4is)
- Adds an evidence-based rationale for recommending NB as part of weight-loss strategies for patients with diabetes and obesity/overweight on an incretin agent as part of the antihyperglycemic regimen.

## Supplementary information

Supplementary Table 1. Absolute Weight Change (kg) from Baseline, Total Population, from Baseline to Weeks 8, 16, 26 and 52
