# Supplementary material for: Extended-release naltrexone/bupropion is safe and effective among subjects with type 2 diabetes already taking incretin agents: a post-hoc analysis of the LIGHT trial
Source: Int J Obes (Lond). 2021 Jun 3;45(8):1687–95. doi: 10.1038/s41366-021-00831-4 (PMC8310797; doi:10.1038/s41366-021-00831-4)
Supplement: Supplementary file 1 — Supplementary Table 1. Absolute Weight Change (kg) from Baseline, Total Population, from Baseline to Weeks 8, 16, 26 and 52 [file 41366_2021_831_MOESM1_ESM.docx]

Supplementary Table 1. **Absolute Weight Change (kg) from Baseline, Total Population, from Baseline to Weeks 8, 16, 26 and 52**

| *Treatment group* | *Week* | *Mean Weight Change* | *Standard Error/* | *Confidence limits* | |
| --- | --- | --- | --- | --- | --- |
|  |  |  |  | *Lower* | *Upper* |
| NB + DPP-4i (n=345) | 8 | -3.47 | 0.14 | -3.75 | -3.18 |
|  | 16 | -4.80 | 0.22 | -5.23 | -4.36 |
|  | 26 | -5.52 | 0.32 | -6.15 | -4.89 |
|  | 52 | -5.89 | 0.45 | -6.76 | -5.01 |
| NB + GLP-1RA (n=339) | 8 | -3.63 | 0.15 | -3.92 | -3.34 |
|  | 16 | -5.16 | 0.23 | -5.61 | -4.72 |
|  | 26 | -5.86 | 0.33 | -6.50 | -5.22 |
|  | 52 | -5.38 | 0.45 | -6.26 | -4.51 |
| PL + DPP-4i (n=317) | 8 | -1.05 | 0.15 | -1.34 | -0.77 |
|  | 16 | -1.64 | 0.22 | -2.08 | -1.20 |
|  | 26 | -1.17 | 0.36 | -1.87 | -0.47 |
|  | 52 | -0.88 | 0.54 | -1.94 | 0.18 |
| PL + GLP1 (n=316) | 8 | -0.82 | 0.14 | -1.10 | -0.53 |
|  | 16 | -1.16 | 0.22 | -1.59 | -0.72 |
|  | 26 | -0.04 | 0.37 | -0.77 | 0.69 |
|  | 52 | 0.65 | 0.57 | -0.47 | 1.76 |
